# Paroxysmal Hypertension in Congenital Hypothyroidism: An Unusual Case of Pheochromocytoma

**DOI:** 10.7759/cureus.72138

**Published:** 2024-10-22

**Authors:** Patrick Fagan, Conor J McLaughlin, Kimberly L Fugok, Tasha Desai

**Affiliations:** 1 Department of Emergency and Hospital Medicine, Lehigh Valley Health Network/University of South Florida (USF) Morsani College of Medicine, Bethlehem, USA; 2 Department of Emergency and Hospital Medicine, Division of Pediatric Emergency Medicine, Lehigh Valley Health Network/University of South Florida (USF) Morsani College of Medicine, Bethlehem, USA

**Keywords:** adrenalectomy, hypertension, pediatrics, pheochromocytoma, recurrence

## Abstract

Pheochromocytomas are rare tumors arising from the chromaffin cells of the adrenal medulla that result in the secretion of excessive catecholamines. They are an uncommon cause of hypertension in the pediatric population. We present a case of a six-year-old male with hypertension, rapid weight loss, palpitations, excessive sweating, and vomiting. Their previous medical history was significant for congenital hypothyroidism, and upon the emergence of his symptoms, his levothyroxine dosing was reduced to half. Presenting symptoms and initial history were suspicious for thyroid-related disease; however, imaging showed a complex right adrenal mass in the setting of elevated plasma catecholamine levels. A diagnosis of pheochromocytoma was made, and a right adrenalectomy was performed. The patient tolerated the procedure well, with complete resolution of symptoms. Three years later, his symptoms re-emerged and were concerning for tumor recurrence. This case highlights the diagnosis of this rare cause of pediatric hypertension and the importance of remaining vigilant against anchoring bias.

## Introduction

Pheochromocytomas are rare tumors arising from the chromaffin cells of the adrenal medulla [1]. Functional pheochromocytomas result in the secretion of excessive catecholamines, leading to clinical features associated with this condition [2]. Pheochromocytomas can be sporadic or hereditary in origin, often occurring in patients with von Hippel Lindau syndrome, multiple endocrine neoplasia type 2, and neurofibromatosis type 1 [1]. Of note, there is no evidence supporting a heritable link between congenital hypothyroidism and pheochromocytoma. While pheochromocytoma has an estimated incidence rate between 0.5 and 0.8 per 100,000 person-years [3,4], it is a potentially underdiagnosed condition [5]. Clinical symptoms manifest as paroxysmal hypertension, headache, tachycardia, and excessive sweating [1,6]. However, symptoms have such variance that pheochromocytomas have been dubbed “the great mimic,” as the symptoms can closely match panic attacks, alcohol withdrawal, hypoglycemia, hyperthyroidism, or sudden cessation of beta-blockers [7,8].

Biochemical tests indicative of pheochromocytoma include elevated metanephrine and catecholamine and should be followed by imaging studies to locate the tumor [6,9]. Definitive treatment involves surgical resection of the tumor [9]. Lifelong, annual biochemical testing is recommended following treatment to assess for recurrence or metastatic disease [10]. We present a case of a six-year-old male who presented to the emergency department with hypertension, acute weight loss, fatigue, palpitations, vomiting, and excessive sweating and was diagnosed with a pheochromocytoma.

## Case presentation

A six-year-old male with a history of congenital hypothyroidism, autism, and asthma presented to the children’s emergency department (ED) complaining of sudden weight loss, fatigue, night sweats, and decreased oral intake. The patient’s mother reported that he had been experiencing a gradual decline in his oral intake over the prior six months, with a sharply worsening appetite in the preceding month. She reported that the patient was no longer able to tolerate oral intake. During this time, the patient lost 13.6 kilograms, with 9.1 kilograms lost in the last month. The patient’s overall energy declined sharply, and he could barely tolerate short walks around their home. Additional symptoms included restless sleep, fatigue, sensation of racing heart, night sweats, pruritis with hot showers, and frequent vomiting with oral intake. They denied fevers, chills, shortness of breath, chest pain, abdominal pain, diarrhoea, and dysuria. Per chart review, the patient’s levothyroxine was decreased from 88mcg to 44mcg daily when symptoms first started, six months prior to presentation.

During the initial physical examination, the patient was hypertensive at 240/168 mmHg, tachycardic at 133 beats per minute (bpm), afebrile (36.6 ^o^C), and had a normal respiratory pattern. He was calm and cooperative but appeared dehydrated with dry mucous membranes. His lungs were clear to auscultation, and his abdomen was soft and non-tender, without notable masses. No focal neurologic deficits were noted. Point-of-care glucose was 207 mg/dL. The patient was treated with 20 mL/kg of normal saline, resulting in improved hypertension to 143/92 mmHg, but tachycardia persisted (143 bpm).

Initial ED lab analyses found hypokalemia at 2.9 mmol/L, hyponatremia at 126 mmol/L, hypochloremia at 87 mmol/L, leukocytosis at 14.0 Th/cm, hemoglobin at14.6 g/dL, and venous blood gas pH of 7.48 with a pCO_2_ of 45 mmHg. Thyroid stimulating hormone was found to be mildly elevated at 4.03 μIU/mL with a free T4 of 1.50 ng/dL and T3 of 4.13 pg/mL (Table 1). Pediatric endocrinology was consulted due to concerns for possible thyroid storm and advised avoiding further medical therapies such as steroids.

**Table 1:** Laboratory analyses performed during the patient’s initial emergency department visit Free T4: free thyroxine, Free T3: free triidothyronine, pCO2: partial pressure of carbon dioxide.

Laboratory Result	Result Value	Reference Range
Potassium	2.9 mmol/L	3.5 – 5.2 mmol/L
Sodium	126 mmol/L	135 – 145 mmol/L
Chloride	87 mmol/L	100 – 109 mmol/L
White Blood Cell Count	14.0 thou/cmm	3.8 – 10.4 thou/cmm
Hemoglobin	14.6 g/dL	11.5 – 15.5 g/dL
Venous pH	7.48	7.31 – 7.41
Venous pCO_2_	45 mmHg	41 – 51 mmHg
Thyroid Stimulating Hormone	4.03 μIU/mL	0.66 – 3.90 μIU/mL
Free T4	1.50 ng/dL	0.81 – 1.35 ng/dL
Free T3	4.13 pg/mL	2.30 – 4.20 pg/mL

**Figure 1:**
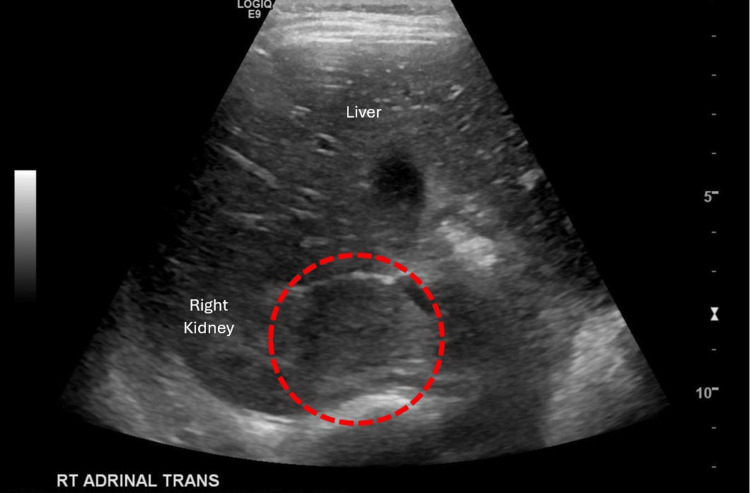
Abdominal ultrasound still image demonstrating 5.3 x 3.3 x 4.0 cm right suprarenal mass (red circle).

Despite initial suspicion of thyroid-related causes for the patient’s symptoms, his episodic hypertension prompted an abdominal ultrasound, which found a 5.3 x 3.3 x 4.0 cm suprarenal mass on the right kidney and a suspected enlarged periportal lymph node (Figure 1). He was admitted to the pediatric intensive care unit (PICU) for further management.

**Figure 2:**
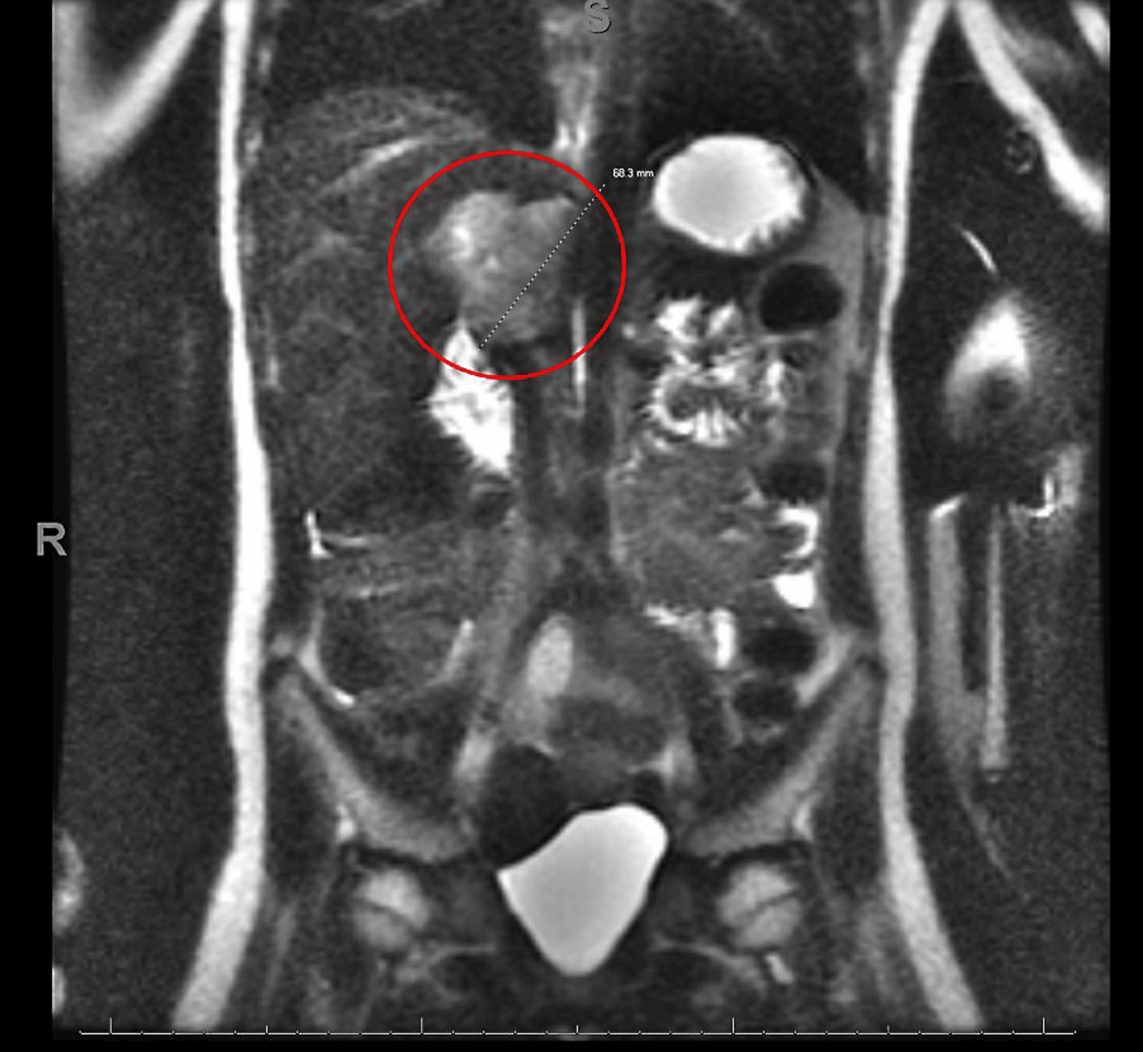
Coronal plane MRI view of the patient’s abdomen. The mass is highlighted by a red circle.

**Figure 3:**
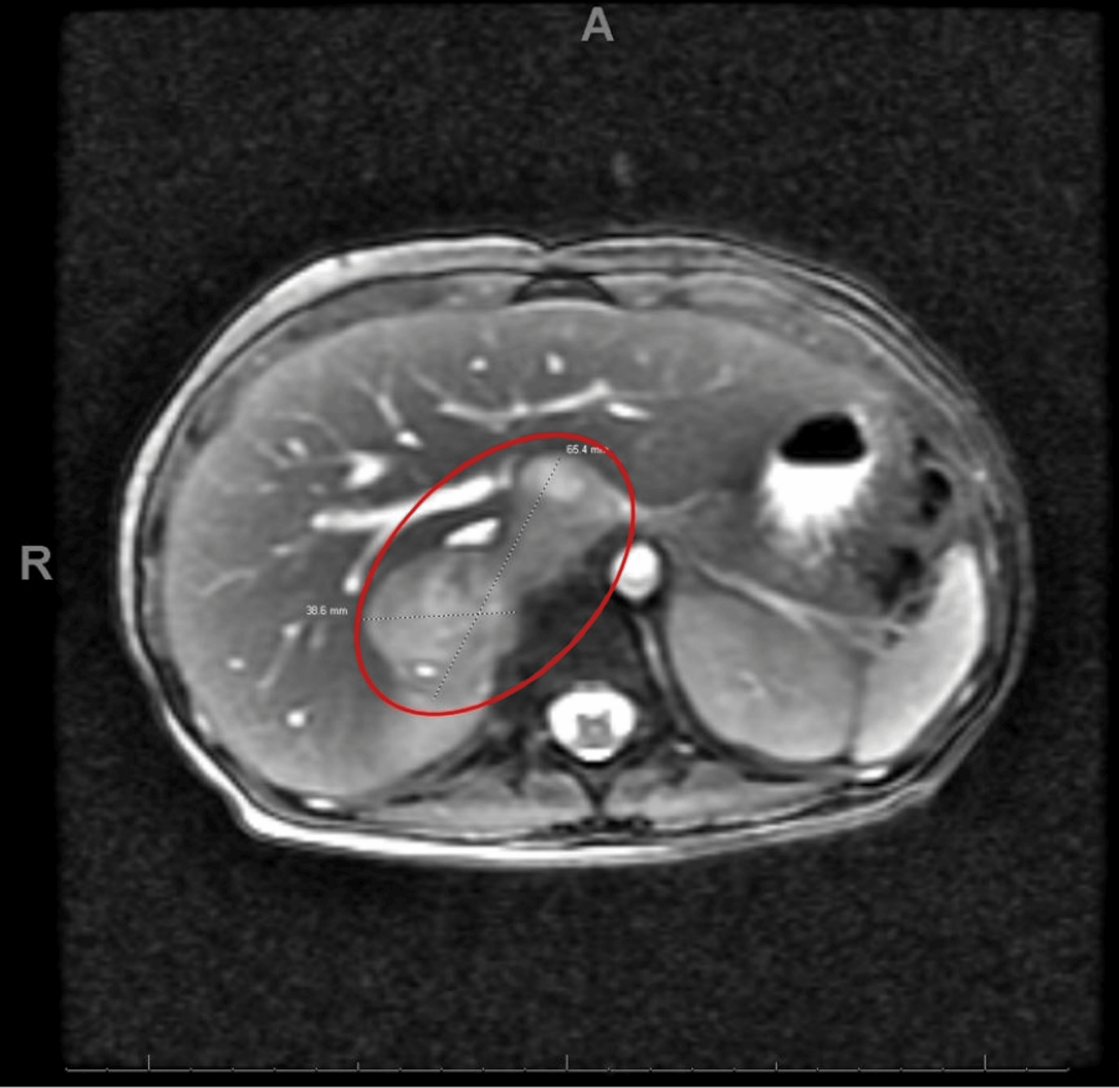
Axial plane MRI view highlighting the suspected pheochromocytoma. The mass is highlighted by a red circle.

While hospitalized, additional labs were obtained and found that sedimentation rate, C-reactive protein, and parathyroid levels were within normal limits, random cortisol level 33 ug/dL, plasma free normetanephrine elevated at 15.90 nmol/L, and plasma catecholamines, norepinephrine and dopamine were elevated at 13,002 pg/mL and 88 pg/mL respectively. An MRI of the abdomen with and without contrast demonstrated a complex lobular right adrenal mass with peripheral enhancement concerning a pheochromocytoma or paraganglioma without evidence of metastatic disease (Figures 2, 3).

The combined indications gathered from the patient’s symptomatology, lab results, and imaging findings led to his final diagnosis of pheochromocytoma. While in the PICU, he received intravenous amlodipine and oral phenoxybenzamine for blood pressure control. The patient was discharged after a nine-day hospitalization with no further adverse events and underwent a complication-free right adrenalectomy two months later for definitive care. In subsequent follow-ups, genetic testing found no known markers associated with pheochromocytoma.

For the following three years, he followed up routinely with endocrinology to monitor symptoms and laboratory markers for recurrence of disease. At nine years of age, the patient returned to the hospital again with hypertension, weight loss and headaches consistent with the recurrence of the tumor. He is currently undergoing treatment for a mass consistent with pheochromocytoma recurrence. He was referred to a multidisciplinary team at a regional children’s hospital, and a surgical biopsy noted metastatic pheochromocytoma on pathology. All visible tumor was removed, and he remains under oncologic surveillance for local or regional metastatic recurrence.

## Discussion

We present a case of a six-year-old male with congenital hypothyroidism and intermittent hypertension secondary to a pheochromocytoma. Paroxysmal symptoms, particularly hypertension, are common among patients with pheochromocytoma, potentially occurring in up to half of those affected [8,11]. However, only 2% of pediatric hypertension is caused by pheochromocytomas and paragangliomas [12].

Anchoring bias is a phenomenon in which clinicians rely too heavily on information discovered early in the diagnostic process, disregarding or deprioritizing information obtained later [13]. By anchoring on early information, clinicians may not adjust their diagnoses when pertinent information is discovered later. In this case, the patient’s preexisting congenital hypothyroidism provided a sufficiently plausible etiology for the patient’s symptoms.

This patient’s slightly elevated TSH for age suggests a hypothyroid state, which would typically decrease the effect of the sympathetic nervous system [14]. The initial differential diagnosis did not include catecholamine-secreting tumors due to anchoring on the presence of known thyroid disease and thyroxine treatment, which can present with similar hypertension, tachycardia, weight loss, and vomiting. Notably, the absence of hyperpyrexia, a common symptom of thyroid storm, decreased clinical suspicion for thyroid-related causes for the patient’s symptoms.

There are limited data on the correlation between pediatric thyroid abnormalities and pheochromocytomas outside of multiple endocrine neoplasia (MEN) syndrome, Von Hippel-Lindau syndrome, neurofibromatosis type 1, and familial paraganglioma syndrome [15]. A paraganglioma/pheochromocytoma panel of 12 genes (FH, MAX, MEN1, NF1, RET, SDHA, SDHAF2, SDHB, SDHC, SDHD, TMEM127, and VHL) associated with pheochromocytoma occurrence found no clinically significant mutations, excluding the possibility of MEN syndrome, von Hippel-Lindau, neurofibromatosis type 1, and familial paraganglioma syndrome. Pheochromocytomas are rare, underdiagnosed tumors that should be included in the differential diagnosis for pediatric hypertension. Pheochromocytomas may present with a classic report of episodic headache. However, paroxysmal hypertension may be the dominant sign [8,11]. Genetic testing and lifelong surveillance should be performed in pediatric patients treated for pheochromocytoma, as cardiomyopathies and tumor recurrence occur in 6-18% of patients [16].

## Conclusions

This case highlights the effect anchoring bias can have when considering a diagnostic differential. While it did not impact our patient’s care, knowledge of his congenital hypothyroidism anchored the physician’s differential as the likely cause of his presenting symptoms, and the unknown pheochromocytoma was not considered a likely case. This initial cause was ultimately ruled out by an abdominal ultrasound study performed as part of the recommended diagnostic course for pediatric hypertension. Visualizing the pheochromocytoma during the abdominal ultrasound prompted a shift in likely cause away from the patient’s hypothyroidism. Though rare, pheochromocytoma should be considered when encountering pediatric hypertension.

## References

[1] Gupta PK, Marwaha B (2024). Pheochromocytoma. StatPearls.

[2] Lenders JW, Duh QY, Eisenhofer G (2014). Pheochromocytoma and paraganglioma: an endocrine society clinical practice guideline. J Clin Endocrinol Metab.

[3] Beard CM, Sheps SG, Kurland LT, Carney JA, Lie JT (1983). Occurrence of pheochromocytoma in Rochester, Minnesota, 1950 through 1979.

[4] Ariton M, Juan CS, AvRuskin TW (2000). Pheochromocytoma: clinical observations from a Brooklyn tertiary hospital. Endocr Pract.

[5] Lo CY, Lam KY, Wat MS, Lam KS (2000). Adrenal pheochromocytoma remains a frequently overlooked diagnosis. Am J Surg.

[6] Lenders JW, Eisenhofer G, Mannelli M, Pacak K (2005). Phaeochromocytoma. Lancet.

[7] Manger WM, Gifford RW (1977). Background and importance. Pheochromocytoma, 1st Ed.

[8] Reisch N, Peczkowska M, Januszewicz A, Neumann HP (2006). Pheochromocytoma: presentation, diagnosis and treatment. J Hypertens.

[9] Yip L, Duh QY, Wachtel H (2022). American association of endocrine surgeons guidelines for adrenalectomy: executive summary. JAMA Surg.

[10] Muth A, Crona J, Gimm O (2019). Genetic testing and surveillance guidelines in hereditary pheochromocytoma and paraganglioma. J Intern Med.

[11] Havekes B, Romijn JA, Eisenhofer G, Adams K, Pacak K (2009). Update on pediatric pheochromocytoma. Pediatr Nephrol.

[12] Rao HK, Jain SB (2022). Pediatric endocrine tumors. Textbook of Endocrine Surgery.

[13] Ly DP, Shekelle PG, Song Z (2023). Evidence for anchoring bias during physician decision-making. JAMA Intern Med.

[14] Hegedüs L, Bianco AC, Jonklaas J, Pearce SH, Weetman AP, Perros P (2022). Primary hypothyroidism and quality of life. Nat Rev Endocrinol.

[15] Edmonds S, Fein DM, Gurtman A (2011). Pheochromocytoma. Pediatr Rev.

[16] Beltsevich DG, Kuznetsov NS, Kazaryan AM, Lysenko MA (2004). Pheochromocytoma surgery: epidemiologic peculiarities in children. World J Surg.

